# Insights from international environmental legislation and protocols for the global plastic treaty

**DOI:** 10.1038/s41598-024-53099-9

**Published:** 2024-02-02

**Authors:** Margrethe Aanesen, Julide C. Ahi, Tenaw G. Abate, Farhan R. Khan, Frans P. de Vries, Hauke Kite-Powell, Nicola J. Beaumont

**Affiliations:** 1 Centre for Applied Research, Norwegian School of Economics, Helleveien 30, 5045 Bergen, Norway; 2 Norwegian Research Center (NORCE), Prof.Olav Hanssensvei 15, 4021 Stavanger, Norway; 3 Department of Environmental Science, Aarhus University, Fredriksborgvej 399, 4000 Roskilde, Denmark; 4 Norwegian Research Center (NORCE), Nygårdsporten 112, 5008 Bergen, Norway; 5 Department of Economics, Business School, University of Aberdeen, Aberdeen, Scotland, UK; 6 Marine Policy Center, Woods Hole Oceanographic Institution, 266 Woods Hole Road, Woods Hole, MA 02543-1050 USA; 7 Plymouth Marine Laboratory, Prospect Place, Plymouth, PL1 3HD UK

**Keywords:** Environmental sciences, Environmental social sciences

## Abstract

Plastic pollution has emerged as a global challenge necessitating collective efforts to mitigate its adverse environmental consequences. International negotiations are currently underway to establish a global plastic treaty. Emphasizing the need for solution-orientated research, rather than focusing on further defining the problems of widespread environmental occurrence and ecological impacts, this paper extracts insights and draws key patterns that are relevant for these international negotiations. The analysis reveals that (i) environmental rather than human health concerns have been the predominant driving force behind previous regulations targeting pollutants, and (ii) the decision to ban or discontinue the use of harmful pollutants is primarily affected by the availability of viable substitutes. These two key findings are relevant to the discussions of the ongoing Intergovernmental Negotiating Committee (INC) on the global plastic treaty and underscore the recognition of environmental consequences associated with plastic pollution while emphasizing the need to enhance the knowledge base of potential human health risks. Leveraging the availability of substitutes can significantly contribute to the development and implementation of effective strategies aimed at reducing plastic usage and corresponding pollution.

## Introduction

In March 2022, at the fifth UN Environment Assembly (UNEA-5.2) in Nairobi, 175 UN Member States voted to establish an Intergovernmental Negotiating Committee (INC) with the mandate of agreeing on a legally binding instrument on plastic pollution by 2024. Resolution 5/14 was adopted and titled “End Plastic Pollution: Towards an international legally binding instrument”. Ambitiously supporting this initiative, in August 2022, the High Ambition Coalition to End Plastic Pollution by 2040 was launched, co-chaired by Norway and Rwanda and at the time of writing including 58 member states^1^. However, plastic is not the first global pollutant that societies have encountered. There have been previous examples of international cooperation in regulating other polluting substances. For instance, the 1987 Montreal Protocol regulated ozone-depleting chemical substances such as chlorofluorocarbons (CFCs), while the 2001 Stockholm Convention regulated the production and use of persistent organic pollutants (POPs). The experiences garnered from these and other international environmental agreements (IEAs), and regional/federal environmental legislation hold significant relevance in shaping the discourse surrounding a global plastic treaty. These agreements and legislations were forged in response to pressing environmental concerns, showcasing the international community’s recognition of the need for coordinated efforts to combat pollution.

Preceding the globally coordinated action to end plastic pollution, and in response to the growing concerns surrounding this challenge, numerous countries have already implemented national and regional measures to mitigate plastics, including efforts to (1) Reduce/Avoid—reducing the quantity of plastics entering the system; (2) Replace/Substitute—substituting plastics with alternative materials, including biodegradables and compostables; (3) Recycle/Re-use—implementing design for recycling, increasing collection capacity, scaling-up sorting and mechanical recycling capacity, scaling-up chemical conversion capacity; (4) Dispose—reducing post-collection environmental leakage, including better disposal facilities, and reducing trade in plastic waste (stop exports); (5) Clean up/remediation—in terms of action there has been a particular focus on improving plastic recycling practices, especially regarding packaging materials^2^. The European Union (EU) serves as an exemplary case where two distinct directives have been introduced to tackle the problem of plastic pollution and promote sustainable practices in EU countries. The first directive, enacted in 2015, specifically addresses the issue of plastic bags. The second directive, implemented in 2019, encompasses single-use plastic products, including fast-food containers, and establishes specific targets for reducing their usage. Many African countries have adopted similar policies to reduce pollution originating from single-use plastics, most notably with bans on plastics bags^3^. Behavioral change interventions aimed at reducing the consumption of single-use plastics, particularly straws and plastic bags, have been implemented in the United States as well (see Ref.^4^ for a review). Co-ordinating these national and regional measures, and building and learning from their implementation within the UN intergovernmental instrument is expected to be highly critical.

Given the large number of existing treaties and regulations to combat pollution of the natural environment and harm to human health, there is the potential for the development of the global plastic treaty to learn from the international management of other pollutants. Determining which factors of those previous arrangements led to the abandoned use of a polluting substance, and how this may apply to regulating plastic pollution, is the primary aim of this study. We analyze features of existing regional environmental legislations and multilateral IEAs, covering the economic, political and scientific aspects to determine whether there are common characteristics that are relevant for the ongoing international discussions surrounding the global plastic treaty. We also look into a few specific IEAs exploring factors for their possible success. Other studies have had similar aims. For example, the study by Raubenheimer & McIlgorm^5^ examining whether the Basel and Stockholm Conventions on hazardous waste and POPs respectively can be used as frameworks for a global treaty to reduce impacts of marine plastic litter. Their study performed a qualitative analysis scrutinizing the Convention texts and analysing hypothetical effects or impacts should the same text be applied to regulate marine plastic waste. They conclude that both Conventions are inadequate to manage the entire lifecycle of all plastic applications. Nunez-Rocha & Martinez-Zarzoso^6^ on the other hand, applying difference-in-differences techniques in a panel data framework, find that ratification of the Rotterdam and Stockholm Conventions leads to reduction in trade of hazardous substances from OECD to non-OECD countries. Our approach is somewhat different. For a set of regulated potentially polluting substances we statistically explore correlations between causing various types of harm, having substitutes, and being banned. First, we do this on a general basis, i.e. including regulated substances in general, and next we do it for two specific IEAs: the Stockholm Convention and the Montreal Protocol.

To accomplish our analysis, we constructed a dataset of 217 polluting substances that have been subject to regulation under the US and EU environmental legislation. We employed correspondence analysis (CA)^7^ to enable the identification of key associations in the dataset. Being a descriptive technique, CA uses biplots to vizualise correlations between the factors describing the regulated substances; i.e. environmental harm, human harm, availability of substitutes and whether the substance is banned form use in economic activity . These correlations uncover key patterns that may provide a basis for formulating robust strategies in the context of a global plastic treaty.

Our approach does not claim any causal relationship between the variables analysed, only statistical correlation. This is different from most analyses identifying common factors behind regulation and banning of polluting substances. For example, in a quantitative model of the ratification of 22 IEAs by a total of 192 states, Roberts et al.^8^ demonstrate that a “narrow export base” (a proxy for a disadvantaged position in the world economy) explains 60% of national propensity to sign environmental treaties. A nation’s natural capital, its ecological vulnerability and international environmental NGO memberships had no explanatory power. Closer to our analysis is Romasheva & Ilinova^9^. Their study explores how various political-legal factors impact the deployment of carbon capture and storage (CCS) projects across selected nations. Using the ratio of the number of active projects and the number of postponed or cancelled projects as the dependent variable and the maturity and variety of the political-legal environment across countries as independent variables they demonstrate a significant positive correlation. This means that more mature policy incentives increase the likelihood for active CCS projects relative to postponed and cancelled projects.

Of more qualitative character is the analysis by Undredahl^10^, explaining how media discovery of scientific evidence indicating ecological damage was an important factor for the German ratification of IEAs combatting transboundary air pollution. the fact that stricter environmental legislation could strengthen the competitive edge of major producers of advanced technology is a source of domestic support for Germany’s active role in international environmental diplomacy^10^. The use of explorative techniques, instead of seeking causal relationships, is advantageous when it is not directly clear which correlations exists among a large number of variables, and when there is little theory or empirical evidence suggesting which are dependent and independent variables. Explorative techniques are typically applied to gain a deeper understanding of a research problem, and is a useful tool when dealing with research problems that are previously not properly investigated^11^. Furthermore, within some disciplines, like epidemeological studies^12^, researchers often collect large amount of data and are interested in exploring the relationships among sets of categorical variables. While one possibility is to conduct separate chi-square tests for each pair of variables, which cumbersome and render results difficult to summarize, correspondence analysis offers a thechnique for exploring all variables simultaneously^13^.

## Material and methods

### Construction of the database

The USA and the European Union (EU) stand as global economic powerhouses, significantly influencing production, use, and trade practices worldwide, particularly regarding potentially polluting substances. Their dominant economic positions and extensive global trade networks mean that regulations enacted within these regions have far-reaching implications, often setting benchmarks for global standards. Consequently, the environmental regulation frameworks from the USA and the EU serve as critical reference points in shaping our database for regulated substances. Hence, we utilize the regulatory frameworks of these regions to construct our database for comprehending and overseeing the utilization of potentially polluting substances on a global scale.

We concentrated on potential polluting substances in the form of chemicals, and include only chemicals with a Chemical Abstracts Service (CAS) number. The CAS number is a unique numerical identifier to every chemical substance described in the open scientific literature. It identifies a chemical over its name. While all larger classes of chemical substances have their own CAS number, some sub-classes are not identifiable with such number. Not having a CAS-number makes it more difficult to find information regarding the substance.

In the USA the main source of information has been the EPA Clean Water Act (https://www.epa.gov/laws-regulations/summary-clean-water-act) and the EPA Clean Air Act (https://www.epa.gov/clean-air-act-overview/clean-air-act-text#toc) Both Acts list all chemicals that are regulated according to them. The EU has formulated a series of lists addressing potentially polluting substances according to the origin of the pollutant and the recipient of the pollution. In addition to these two very extensive frameworks, there are a large number of international treaties regulating the production, use, and trade in potentially polluting substances. These include the Stockholm Convention to regulate persistent organic pollutants (POPs), the Rotterdam Convention on hazardous substances, the Helsinki Protocol on (long range) sulphur emissions, and the Montreal Protocol on ozone depleting substances. These are addressing specific pollution problems. They also list the substances that are regulated according to them, and closer inspection reveals that these overlap with the substances included in the US and EU environmental legislation.

Overall, a total of 217 substances were coded with regard to chemical and socio-economic characteristics. Table 1 contains an overview of the environmental legislation used to develop the dataset. Many of the substances are regulated by more than one act/directive/list.

**Table 1:** Overview of regulations used to produce the dataset.

Regulation measure	Year of adoption	No of substances included
EPA clean water act priority substances	1972	119
EPA-clean air act on hazardous substances	1970	76
EU REACH restricted list	2006	32
EU REACH BPR list	2012	3
EU cosmetics directive	1976	77
EU REACH candidate list	2013	29
EU Water framework directive	2000	48
EU POP pollutant	2019	41
EU RoH pollutant	2003	8
EU Annex III	2000	97
EU pre-registered substances	2008	174

The characteristics of the regulated substances cover a large and heterogeneous selection of properties and we have grouped them according to six different criteria, given in the first line of Table 2. The selection of characteristics (variables) was based on available information in open websites. These include “The coding for the chemical characteristics relies on data sourced from reputable institutions, including the US National Library of Medicine (https://pubchem.ncbi.nlm.nih.gov/), Green Screen for Safer Chemicals (greenscreen.org), ContaminantDB by Canadian Institutes of Health Research, Canada Foundation for Innovation, The Metabolomics Innovation Centre (TMIC), and Chemical Hazards and Alternatives Toolbox (chemhat.org). To capture socio-economic characteristics, information is gathered from diverse outlets such as SINLIST and ChemSec Marketplace (International Chemical Secretariat), Substitution Support Portal (SUBSPORTplus) by the German Federal Institute for Occupational Safety and Health, Sigma-Aldrich Market Place, ECHA Substance Info Cards, and the US National Library of Medicine (https://pubchem.ncbi.nlm.nih.gov/).

**Table 2:** Grouping of characteristics: The first line is a common name for the group of characteristics.

Chemical group	Fate of substance	Harmful effects on humans	Environmen-tal impact	Spread of substance	Use in economic activity
Volatile organic compound (VOC)	In food	Carcinogen	Ozone depletion	Uniformly mixing polluter vs. non-uniformly mixing polluter	Substitutes available
Brominated fire retardant (BFR)	In plastic	Human-animal toxic	Climate change	Point source polluter vs. non-point source (diffuse) polluter	Discontinued production
Persistent organic pollutant (POP)	In personal/home products		Acute aquatic toxicity		Unit price
CFC/HCFC/.	In air	Cause mutation in human DNA (Mutagen)	Chronic aquatic toxicity		Volume of production
Polybutylene terephthalate (PBT)	In water	Cause birth defects	Land ecosystem toxicity		
PCB/PCDD/PCDF		Toxic to reproduction			
PAH		Endocrine disruptive activity (Interact with hormones)			

The other lines are the characteristics included in that group.

Substances are regulated either because they are harmful to the natural environment, cause harm to human health, or both. The formulation of the regulation may differ depending on whether the dissemination of the potentially polluting substance is a point source or not, or uniform or not. Hence, crucial information is the type of harm caused by the substance and the type of dissemination. These characteristics we combine with information on how the international society handles potentially polluting substances; are substitutes readily available and how strict the regulations are.

In Table 2, the first set of characteristics provides the classification of the substance depending on the group of chemicals they belong to, where some pollutants may belong to several chemical groupings. The next set of chemical characteristics indicates whether the substance is found in air and/or water, plastics, personal/home products, and whether it is a food toxin. A third set of characteristics is based on scientific evidence regarding the substances’ impact on animal and human health (toxicity, carcinogenicity, mutagenic properties, endocrine activity, reproduction toxicity). Different from the toxicity in aquatic systems, toxicity for humans and animals was defined as a fatality when ingested or inhaled by humans and other mammals, while the impacts on aquatic life can be immediate or chronic. A fourth set of chemical characteristics is based on scientific evidence regarding the substances’ impact on ecosystems (toxicity in aquatic and land ecosystems), climate change, and ozone layer depletion. A fifth set of chemical characteristics indicates to what degree the substance disperses uniformly or not when emitted into the natural environment, and whether it is a point-source, i.e. origins from an identifiable source like a pipe or a drain, or non-point source (diffuse) pollutant.

The socio-economic characteristics involve the stringency of the regulations applied to the substance and the availability of substitutes with less or no harmful impacts. In addition, for some substances price of the substance, and annual production volumes in the EU and US are given. The “availability of substitutes” is a variable that indicates whether the polluting substance can be substituted with a more environmentally friendly substance when used as input in economic activity. While there may be fewer polluting alternatives that perform the regulated substance’s main functions adequately, it may also be that they fail to imitate other functions in the production process. Hence, the presence of substitutes does not necessarily imply “perfect” substitutability over multiple functions. As it is difficult to distinguish between how well substitutes cover all or only parts of the functions of the regulated substance, we only distinguish between whether substitutes exist or not. The same is the case for regulatory strictness, where the only distinction is between whether a regulated substance is abandoned when it comes to use in economic activities or not.

### Statistical analysis

Correspondence analysis (CA), a multivariate statistical technique applied to categorical and binary variables, transforms any data table (matrix) into contingency table(s) to analyse linear combinations of sets of variables that maximize the variation contained within them^7^. CA uses the chi-square distance based on the Pearson’s residuals. As it is a descriptive technique, it can be applied to tables regardless of a significant chi-square test. In our data, the rows represent the substances and the columns the factors characterizing them, and the chi-square distance measures the similarity of regulated substances with respect to selected factors. The outcome can, by the use of biplots, visualize any structure hidden in the multivariate setting of the table. Points close to each other represent rows/columns with very similar values in the original data. Columns with similar profiles are grouped together in the biplot, and negatively correlated columns are located on opposite sides of the plot origin.

For each chemical in our dataset 12 factors were used for analysis within the categories of environmental harm (4 factors), Human harm (6 factors) and socio-economic factors (2 factors) (Table 3). The four factors used to describe environmental harm for the chemicals were: contribution to climate change (CC), contribution to the ozone layer depletion (OZON), and the exposure to the chemicals causing toxicity to aquatic organisms (AQUA_TOX) or terrestrial organisms (LAND_TOX). Six factors were used to describe human harm for each chemical: carcinogenic (CARC), mutogenic (MUTAG), endocrine disruption (ENDA), reproductive toxicity (i.e. reduced fertility (REP_TOX), teratogenic (ie. causing birth defects (BIRTH)) and lastly one additional factor to describe toxicity to humans (HA_TOX) in ways other than those already mentioned. Environmental and human harm factors are ordered categorical variables, and for each chemical a score was assigned per factor between 0 and 4, where 0 means no effect and 4 means considerable effect.

**Table 3:** Overview of variables included in the correspondence analysis.

Categorical variables (scored 0–4)		Dummy variables (scored 0 or 1)
Environmental harm factors	Human harm factors	Socio-economic factors
Contribution to climate change (CC)	Causing cancer (CARC)	Regulatory stringency (BAN)
Harm to the ozone layer (OZON)	Mutation in DNA (MUTAG)	Availability of substitutes (SUBS)
Toxicity to aquatic organisms (AQUA_TOX)	Toxicity to humans (HA_TOX)	
Toxicity to land organisms (LAND_TOX)	Harmful to reproduction (REP_TOX)	
	Interfering with hormones (ENDA)	
	Causing birth defects (BIRTH)	

The environmental and human harm factors were combined with two dummy variables expressing regulatory stringency and the availability of substitutes when used in economic activity. With respect to regulatory stringency the value 1 is assigned if the substance is abandoned for use in economic activity (BAN) and zero otherwise. With respect to substitutes, the value 1 is assigned in case substitutes are available (SUBS) for use in economic activity and zero otherwise. This coding was made in accordance with the regulatory guidelines and substance property definitions found in prominent inventories such as ChemSec SINLIST, Chemical Hazard and Alternatives Toolbox (ChemHAT), ContaminantDB, European Chemicals Agency (ECHA), and PubChem. These resources offer comprehensive insights into the environmental and health hazards associated with various substances, along with information on corresponding regulatory requirements and the availability of potential substitutes.

The factors presented in Table 3 are given by columns in a matrix, and the 217 regulated substances are given by the rows. CA transforms this matrix into a contingency table using the chi-square distance^7^, breaks down the total variation in the data, and presents it by “dimensions” (corresponds to principal components (PCs) in PCA), where each dimension corresponds to the amount of variation retained as measured by the Eigenvalue^14^. The more heterogeneous the data is, the more dimensions are needed to retain the total variation. In the biplots, each axis represents one dimension. The factors’ relative location in the biplots indicates to what degree they discriminate between the substances; the further a factor is located from the origin the larger the variation among substances with respect to this factor. Furthermore, along each Dim the closer two or more factors are located, the more equal are the substances with respect to these factors. The statistical measure for the association between a variable (column) and a Dim, is the squared cosine (cos2), also called quality of representation. If a variable is well represented by the two dimensions in a biplot, the sum of the cos2 is close to 1. In our biplots this is also indicated by color, where blue colors indicate a high quality of representation whereas red colors indicate a low quality of representation.

All statistical models were run in RStudio version 4.2.0, using the following packages; FactoMineR, factoextra, dplyr, tidyverse, gplots and corrplots.

## Results

Our analysis yielded three main results: (i) the decision to strictly regulate pollutants is primarily influenced by the availability of viable substitutes rather than the scientific weight of evidence that they cause harm to the environment or humans; (ii) evidence for environmental concerns have been a key driving force behind previous pollution regulations, prioritizing the mitigation of environmental harm over potential risks to human health; and (iii) the Montreal Protocol, designed to protect the stratospheric ozone layer by phasing out CFCs, serves as a noteworthy model for transitioning away from polluting substances that resonate with the current state of process related to plastic pollution.

### Environmental harm

**Figure 1:**
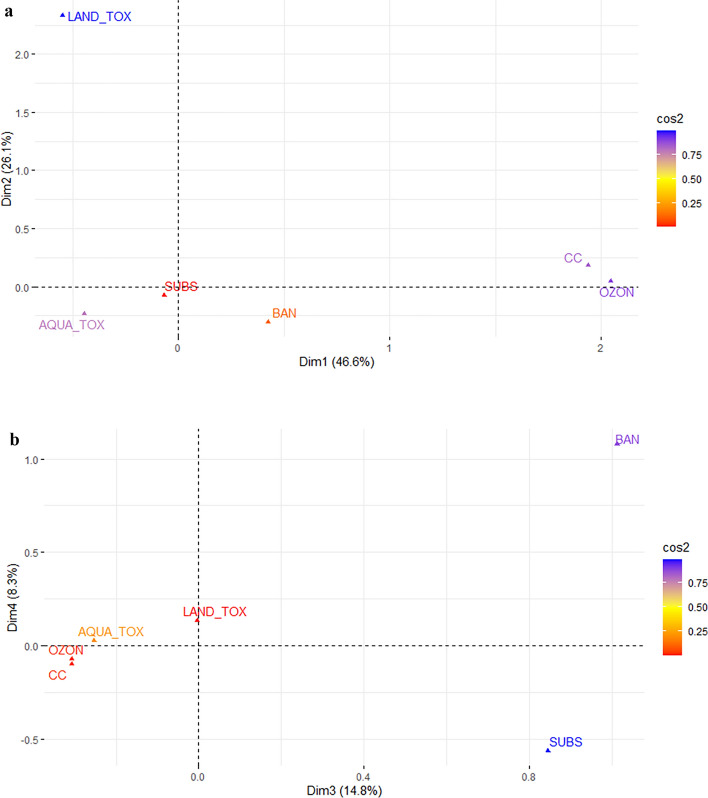
Biplots for CA of the factors indicating which environmental harm the substances cause and whether they are banned or not or have close substitutes or not. 209 out of 217 substances included. Dim 1 and 2 in panel (**a**), Dim 3 and 4 in panel (**b**). The color bar to the right shows the cosine square (cos2), and higher numbers (blue colors) indicate that the variable is well represented in the factor map.

Figure 1 presents results from the CA on a matrix combining the environmental harm and socio-economic factors displayed in Table 3 and including 209 out of 217 substances (substances with 0 score on all factors are deleted). While it is possible to display both factors and the substances in the biplot, we concentrate on the factors and depress the regulated substances because including the 209 substances makes the biplot difficult to read. In the case of environmental harm, it takes five dimensions (Dim) to explain 100% of the variation in the data. Each Dim corresponds to the proportion of variance covered, as measured by the Eigenvalue, and the first Dim account for the largest proportion, Dim 2 the second largest, and so on. Figure 1 displays the first four Dim, which cover 95.7% of the total variation in the dataset. These four Dim are displayed along the x- and y-axes in the biplots in Fig. 1. In the supplementary information, Fig. S1, is the biplot of Dim 4 and 5.

Panel A of Fig. 1 displays Dim 1 and 2, representing 72.7% of total variation, and the color of the factor names shows that LAND_TOX contributes the most to explain variation along Dim 1 and 2, whereas SUBS and BAN explain the least of variation along the two first Dim. This can be seen from the color bar to the right in the plots giving the relative contribution of the factors to the two Dim. Dim 1 (x-axis) shows that substances contributing to climate change (CC) and ozone layer depletion (OZON), are closely associated with each other and that substances causing land and aquatic toxicity (LAND_TOX and AQUA_TOX, respectively) are closely associated with each other. Further, the fact that (CC & OZON) and (LAN_TOX & AQUA_TOX) are located at opposite ends of the x-axis means that substances vary greatly with respect to scoring on the two former and the two latter. Dim 2 (y-axis) explains variation in the data when variation along Dim 1 is controlled for. Dim 2 shows that LAND_TOX stands out from the other factors, i.e. the substances differ greatly when it comes to whether they are toxic to land ecosystems but not when it comes to the other factors. The colors confirm LAN_TOX is the single factor that explains most of the variation in the biplot, whereas SUB and BAN explain the least variation.

Panel B of Fig. 1 displays Dim 3 and 4, representing 21.3% of the total variation in the data, and from the color bar we can see that it is the regulations stringency (BAN) and presence of substitutes (SUBS) that explain the most of variation along these Dim. Dim 3 (x-axis) shows that substances being banned and having substitutes are closely correlated, and these distinguish from substances that cause ozone depletion, climate change and are toxic to aquatic ecosystems. Dim 4 (y-axis) shows that when variation along the three first Dim are controlled for, it is regulation stringency (BAN) that stands out from the other factors, i.e. the substances differ greatly when it comes to whether they are banned as input in economic activity, but less when it comes to the other factors. In particular, Dim 4 distinguishes between the factors BAN and SUBS as the two extremes, i.e. those substances being banned for use in economic activity are the least associated with substances that have substitutes when used in economic activity. Combining Dim 4 and 5 (see Supplementary information Fig. S1) confirms that Dim 4 distinguishes regulatory stringency (BAN) from the other factors, while Dim 5 mainly distinguishes between harming the ozone layer (OZON) and causing climate change (CC). Dim 5 shows that all factors except for CC and OZON are closely related. The colors confirm that SUBS and BAN are the factors that explain most of the variation in the biplot.

While the biplots visually describe associations between characteristics of regulated substances, it does not indicate the degree of statistical significance of the correlation. The significance of the correlations among variables in the model can be tested by Pearsons chi-squared test of statistically significant difference between expected frequencies and observed frequencies in one or more categories of a contingency table. For the biplot above the test statistic equal 1278, with a p-value equal to 0.000. Hence, we reject the null hypothesis (H0) about independence, which means that the regulated substances differ significantly when it comes to the distribution of values on the characteristics. In turn, this means that the distance between the factors in the biplots is statistically significant.

### Human harm

**Figure 2:**
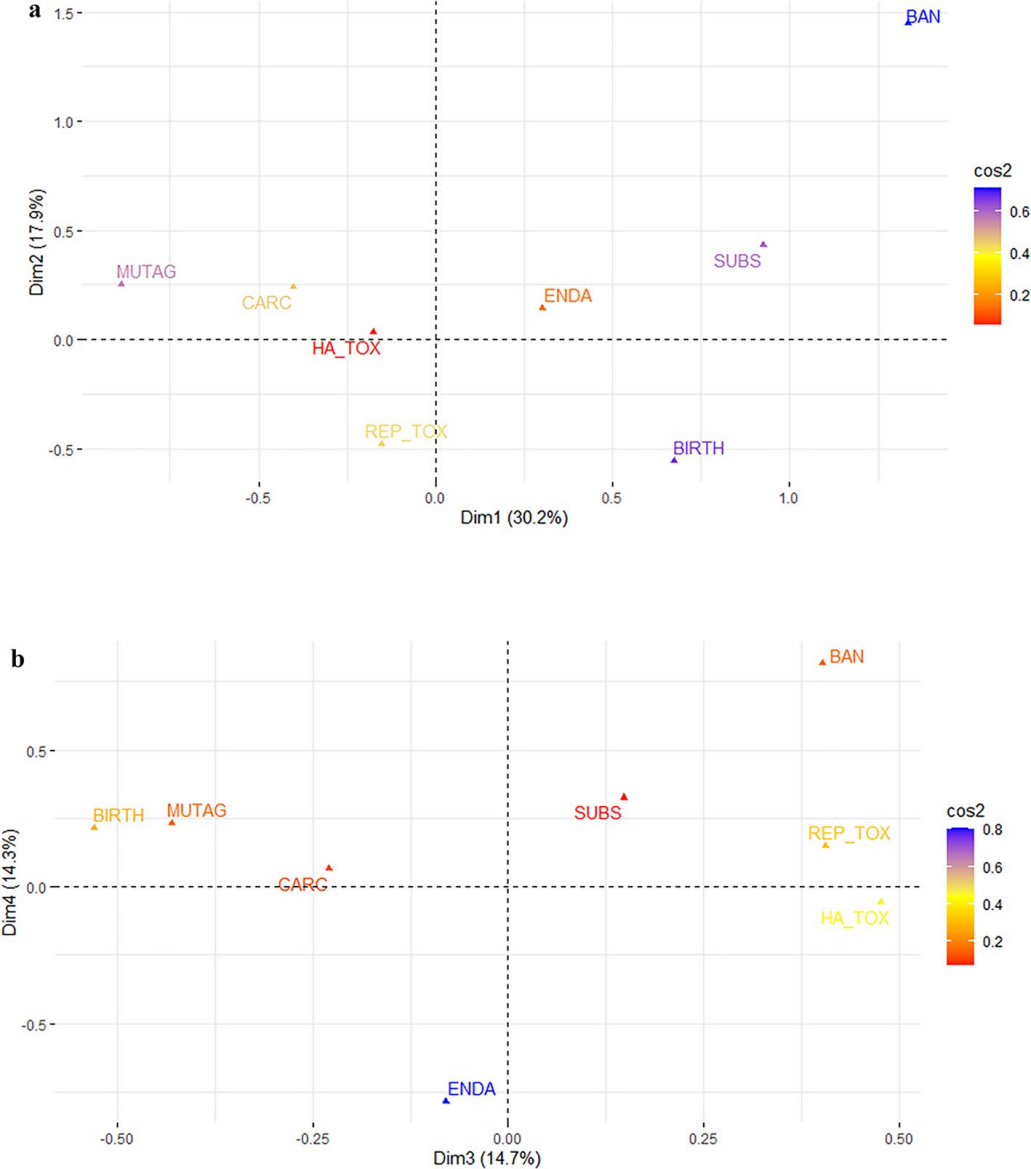
Biplot for CA of the factors indicating which human harm the substances cause and whether they are banned or not or have close substitutes or not. 217 substances included. Dim 1 and 2 in panel (**a**), Dim 3 and 4 in panel (**b**). The color bar to the right shows the cosine square (cos2), and higher numbers (blue colors) indicate that the variable is well represented in the factor map.

Figure 2 presents results from CA of data combining human harm and socio-economic factors (see Table 3). The total variation in this data is explained by a total of 7 Dim. While Fig. 2 displays biplots representing the first 4 Dimensions, biplots for Dim 5 and 6, and 6 and 7, can be found in the supplementary Figs. S1 and S2. Panel A of Fig. 2 displays Dim 1 and 2, representing 48.1% of the total variation, and the color bar to the right shows that it is BAN and BIRTH that contribute the most to explain variation along Dim 1 & 2, whereas HA_TOX explain the least of the total variation. Dim 1 (x-axis) shows that being banned (BAN) and causing mutation in DNA (MUTAG) are the factors that discriminate the most among the substances. In other words, for a particular substance, there is little correlation between causing mutation in DNA and being banned. Dim 2 (y-axis) shows that when variation along Dim 1 is controlled for, it is BAN and BIRTH that discriminate the most among the substances, i.e. there is little correlation between substances causing birth defects and the fact that they are banned. The colors confirm that BAN is the single factor explaining most of the variation, and that BIRTH, SUBS and MUTAG also contribute well to explain variation in the biplot.

**Figure 3:**
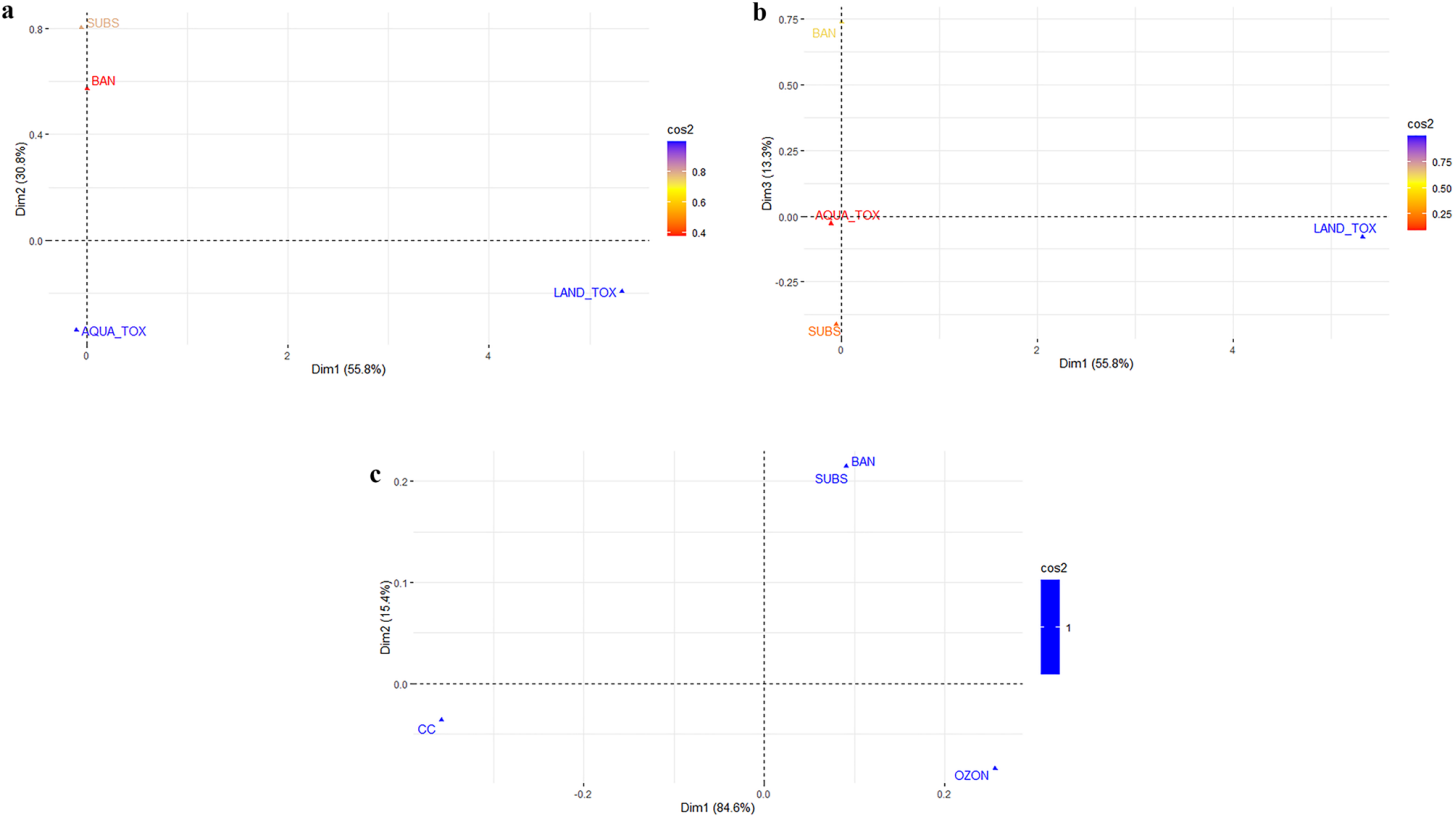
Biplot for CA of factors indicating environmental harm and whether they are banned (BAN) and have close substitutes (SUBS) for substances regulated by the Stockholm Convention (panel (**a**) and (**b**), N = 71) and the Montreal Protocol (panel (**c**), N = 16). All Dim included. The color bar to the right shows the cosine square (cos2), and higher numbers (blue colors) indicate that the variable is well represented in the factor map.

Panel B of Fig. 2 displays Dim 3 and 4, representing 29% of the total variation, and the color bar to the right indicates that it is the factor endocrine disruptive activity (ENDA) that explains most of the variation along these Dim, whereas SUBS contribute the least. Dim 3 (x-axis) shows that substances being toxic to reproduction (REP_TOX) and toxic (HA_TOX) are closely correlated, and these distinguish significantly from substances that cause birth defects (BIRTH) and mutations in DNA (MUTAG). Moreover, substances being toxic to reproduction and to humans in general are also closely correlated with being banned. Dim 4 (y-axis) shows that when variation along the three first three Dim is controlled for, substances that are banned are the least associated with substances being endocrine disruptive (ENDA). Colors show that ENDA is the single factor contributing the most to explaining the variation accounted for in this biplot.

Along all the first 4 Dim the factor SUBS, i.e. whether a substance has close substitutes when used in economic activity, is the factor that is closest associated with BAN, i.e. whether a substance is banned from use in economic activity. This is true although the association along Dims 2, 3 and 4 is not very close. In the supplementary information, Fig. S2 shows that this is no longer the case in Dims 5 and 7. Along these two Dims being banned from use in economic activity (BAN) is closer related to ENDA (interfering with hormones) and with REP_TOX (being toxic to reproduction), CARC (causing cancer) and MUTAG (causing mutation in human DNA). Dim 7 and 5 account for about 15% of the total variation in the data.

The chi-square test statistic for the model equal 2055 (p-value = 0.000). Hence, we reject the H0 about independence, which means that the regulated substances differ significantly when it comes to the distribution of values on the characteristics. In turn, this means that the distance between the factors in the biplots is statistically significant.

There are some interesting lessons from the CA results above. Comparing results from Figs. 1 and 2 we can see that while being banned or having close substitutes when used in economic activity are not important factors in discriminating between substances causing environmental harm (Fig. 1), these two factors do discriminate between substances causing human harm (Fig. 2). In other words, substances causing environmental harm tend to be similar when it comes to being banned and having substitutes, and closer inspection shows that they are likely to score positive on both. Substances causing human harm, on the other hand, tend to differ substantially when it comes to being banned and having substitutes. Hence, while being banned and/or having close substitutes is a unifying factor for substances that cause environmental harm, this is not the case for substances that cause human harm. Furthermore, the factors BAN and SUBS are the two factors closest associated along all Dims except for Dim 4, accounting for 8% of total variation when they are combined with environmental harm factors. The corresponding numbers when combined with human harm factors are 2 (Dim 5 and 7), accounting for 15% of total variation. Hence, there is a closer association between being banned from use in economic activity and having substitutes when used in economic activity for substances causing environmental harm compared to substances causing human harm.

### The global agreements

Next, we focus on the Stockholm Convention and the Montreal Protocol that were aimed at regulating substances causing climate or environmental harm. Figure 3 displays CA biplots capturing the relevant environmental harm and socio-economic factors of the substances included in each of the two agreements, respectively. Panels A and B in Fig. 3 show results for the substances regulated under the Stockholm Convention and Panel C relates to substances within the the Montreal Protocol. Panels A and B display 100% of the variance in the data including the Stockholm Convention substances, whereas the Panel C biplot displays 100% of the variance in the data included within the Montreal Protocol substances.

The chi-square test statistic for the model of the Stockholm Convention substances equal 206 (p-value = 0.56). Hence, we cannot reject the H0 about independence, which means that the regulated substances do not differ significantly when it comes to the distribution of values on the characteristics. In turn, this means that the distance between the factors in the biplots is not statistically significant. For the model of the Montreal Protocol substances the chi-square test statistic equal 72.66 (p = 0.55), and the same conclusions as for the Stockholm Convention substancs apply.

The results in panel C of Fig. 3, representing results from the CA of substances included in the Montreal Protocol (rows) and environmental harm and socio-economic factors (columns) are interesting. First, from the color bar to the right we can see that all factors contribute equally in explaining the variation in the data, and total variation is explained by only two Dim. The fact that the factors SUBS and BAN coincide means that they take the same value for all substances, i.e., if a substance has substitutes when used in economic activity it is also banned, and vice versa. Dim 1 (x-axis) demonstrates that the most important variation is between substances causing depletion of the ozone layer and substances causing climate change. Substances causing depletion of the ozone layer are closer to the factors SUBS and BAN, indicating that these substances are more likely to have substitutes and be banned relative to substances causing climate change. This makes sense since it was the ozone-depleting substances the Montreal Protocol was designed to regulate. Dim 2 (y-axis), accounting for only 15% of total variation, demonstrates some variation between having substitutes and being banned on the one hand, and causing depletion of the ozone layer and climate change on the other hand. This variance accounts for the fact some substances causing climate change do not have substitutes and/or are banned.

Panels A and B in Fig. 3 display results from the CA on data including substances regulated under the Stockholm Convention (rows) and environmental harm and socio-economic factors (columns). Different from the Montreal Protocol substances, there is no overlap between being banned (BAN) and having substitutes when used in economic activity (SUBS), which means that substances that have substitutes are not necessarily banned, and vice versa. In other respects the results are similar to those for the Montreal Protocol substances. The Stockholm Convention substances differ mainly when it comes to the factor LAND_TOX, whereas they are relatively similar when it comes to the factors AQUA_TOX, SUBS and BAN. The latter means that causing harm to aquatic ecosystems (AQUA_TOX) is highly correlated with being banned and having substitutes. However, Dim 2 and 3, accounting for about 45% of the total variation, demonstrate that there are distinctions between being banned and having substitutes on one hand, and being toxic to aquatic environments on the other, and between having substitutes and being banned. Hence, although the Stockholm Convention is also effective in addressing the substances it has set out to regulate there is not an equally strong correlation between having substitutes, being banned and being toxic to the aquatic environment.

## Discussion

Our results demonstrate that historically, polluting substances are more likely to be banned when they are associated with environmental harm compared to human harm. During the last two decades, a substantial body of research has consistently demonstrated that plastics exert a detrimental impact on the environment^15–18^. Hence, the fact that our knowledge regarding the impacts of plastics on human health is limited should not impede the establishment of a regulatory instrument focused on addressing plastic-related pollution.

Furthermore, our results provide evidence that the decision to ban the use of specific polluting substances is strongly associated with the availability of viable alternatives. The successful substitution of substances within the framework of the Montreal Protocol serves as a noteworthy example of transitioning away from detrimental materials in production and consumption. Acquiring a comprehensive understanding of available substitutes, and effectively leveraging this knowledge, can significantly contribute to the formulation and implementation of strategies aimed at curtailing plastic consumption and mitigating the associated polluting impacts. Plastic substitutes have gained significant attention as potential alternatives to traditional fossil-fuel based plastic materials^19,20^. These substitutes offer both advantages and drawbacks, which warrant a careful evaluation for informed decision-making. On the positive side, many plastic substitutes are derived from renewable resources such as plant-based materials, reducing the reliance on fossil fuels. Furthermore, certain substitutes exhibit biodegradable features, allowing for more efficient decomposition and reducing their persistence in the environment. Additionally, some substitutes offer enhanced recyclability and can be integrated into existing waste management systems more effectively.

However, plastic substitutes also present certain disadvantages in the form of a higher carbon footprint and biodiversity loss. Life-cycle analyses have revealed that manufacturing processes and transportation requirements for some substitutes may result in increased greenhouse gas emissions compared to Conventional plastics^21–24^. Moreover, the widespread adoption of certain substitutes such as paper may necessitate significant land and water resources for cultivation and processing, potentially leading to environmental concerns such as deforestation or water scarcity. Plastic substitutes may also be more expensive than traditional plastics due to factors such as limited availability, higher production costs, or the need for specialized equipment and technologies^25,26^. As economies of scale are realized and production methods become more streamlined, the prices of substitutes will likely decrease over time, reinforcing their economic viability.

It is recognized that public awareness and the support of influential persons can contribute to the successful implementation of IEAs, and the Montreal Protocol is often used as the ultimate example of such a success story^27^. However, a critical review of the history of the Montreal Protocol^28^ demonstrates the strong role of the chemical industry, and atmospheric scientists hired by them, in facilitating the phase-out of CFCs. Gareau^28^ also argues that the phasing out of CFCs would have experienced significant setbacks had alternatives to CFCs been considerably more expensive, or if scientific knowledge regarding alternatives to CFCs had not been close to the implementation stage. This does not imply that civil society pressure did not play a role in pushing the U.S. government to phase out CFCs, even in the absence of such abandoning from other industrial countries. But their influence succeeded mainly in the area where the economic consequences were the smallest, that is, the production and use of aerosols^28^. This finding is particularly pertinent in the context of plastics, as a substantial lobby exists that advocates for addressing plastic-related issues^29,30^. Furthermore, public opinion towards plastic pollution exhibits a predominantly negative disposition. Research has consistently demonstrated that consumers tend to perceive plastics as environmentally detrimental, irrespective of the actual environmental characteristics of alternative packaging materials^31^.

Bellelli et al.^32^ highlight the impact of income disparities on a country's capacity to participate in IEAs. Therefore, it is crucial for a global plastic treaty to incorporate provisions that facilitate the involvement of developing nations. In this regard, the Montreal Protocol provides a valuable lesson, as it was the first IEA to successfully implement an effective financial mechanism. The financial mechanism allowed monetary transfers across developing and developed countries to ensure that no single country needed to bear unreasonably large financial burdens by ratifying the Protocol. Hence, the Protocol took into account the diverse economic capabilities and environmental concerns across nations. By considering the specific needs and circumstances of developing nations, the Montreal Protocol fostered a sense of ownership and participation, thereby promoting a more equitable and effective implementation^33,34^. Drawing from this, a global plastic treaty should adopt a similar composition mechanism that ensures the meaningful participation of all nations, particularly those with limited financial resources. Such provisions can help bridge the income disparities and address the unique challenges faced by developing countries in tackling plastic pollution. By facilitating active involvement, the treaty can benefit from a diversity of perspectives, expertise, and financial support, thereby enhancing its overall effectiveness and global impact.

## Conclusions

By exploring common characteristics of regulated substances in existing environmental legislation in major economies we show that the burden of proof relating to environmental harm is more strongly associated with the passing of stricter regulations compared to the demonstration of harm to humans. Furthermore, regulated substances that have substitutes when applied in economic activity are more associated with being regulated strictly, (i.e. banned) compared to substances with no such alternatives. This is the case even for relatively “weak” substitutes where the alternative substances do not entirely match the task of the regulated substance.

Our results based on general environmental legislation in leading economies are supported by closer inspection of two IEAs, namely the Montreal Protocol and the Stockholm Convention. Similar statistical exploration of characteristics of substances regulated by these IEAs confirms the close association between substances that are banned for use in economic activity and substances having (close) substitutes when applied in economic activity.

Regarding the ongoing Intergovernmental Negotiating Committee (INC) on the Global Plastics Treaty, there is a weight of scientific evidence documenting environmental presence and harm, and much ongoing research into the development and validity of “benign by design” alternatives. Based on previous successful IEAs, highlighting these two specific areas, will lead to a more strict regulation being applied to plastics.

### Supplementary Information

Supplementary Information.

## Data Availability

The dataset has been developed by the authors and is accessible from the corresponding author on request. A readme file can be found along with the codes in GitHub providing names, descriptions and type of coding of the pollutants. When results from the analysis are published the dataset will be uploaded in an open data repository.

## Code availability

The code used for producing the results presented in this paper is available on GitHub; https://github.com/maanesen/marineplastic.
