# Author Correction: Insights from international environmental legislation and protocols for the global plastic treaty

**DOI:** 10.1038/s41598-024-57568-z

**Published:** 2024-03-25

**Authors:** Margrethe Aanesen, Julide C. Ahi, Tenaw G. Abate, Farhan R. Khan, Frans P. de Vries, Hauke Kite-Powell, Nicola J. Beaumont

**Affiliations:** 1 Centre for Applied Research, Norwegian School of Economics, Helleveien 30, 5045 Bergen, Norway; 2 Norwegian Research Center (NORCE), Prof.Olav Hanssensvei 15, 4021 Stavanger, Norway; 3 Department of Environmental Science, Aarhus University, Fredriksborgvej 399, 4000 Roskilde, Denmark; 4 Norwegian Research Center (NORCE), Nygårdsporten 112, 5008 Bergen, Norway; 5 Department of Economics, Business School, University of Aberdeen, Aberdeen, Scotland, UK; 6 Marine Policy Center, Woods Hole Oceanographic Institution, 266 Woods Hole Road, Woods Hole, MA 02543-1050 USA; 7 Plymouth Marine Laboratory, Prospect Place, Plymouth, PL1 3HD UK

Correction to: *Scientific Reports* 10.1038/s41598-024-53099-9, published online 02 February 2024

The Acknowledgements section in the original version of this Article was omitted. The Acknowledgements section now reads:

“This study was supported by NAMC -North Atlantic Microplastic Centre. Pillar 5 "Society and Regulation", 2021–2023, https://namc.no/about.”

The original Article has been corrected.

