# Supplementary material for: Insights from international environmental legislation and protocols for the global plastic treaty
Source: Sci Rep. 2024 Feb 2;14:2750. doi: 10.1038/s41598-024-53099-9 (PMC10834483; doi:10.1038/s41598-024-53099-9)
Supplement: Supplementary file 1 — Supplementary Information. [file 41598_2024_53099_MOESM1_ESM.docx]

Supplementary information

Manuscript: **Insights from Past International Protocols and legislation for the Global Plastic Treaty**

Corresponding author: Margrethe Aanesen; Margrethe.aanesen@snf.no

**Supplementary figure S1:**

#
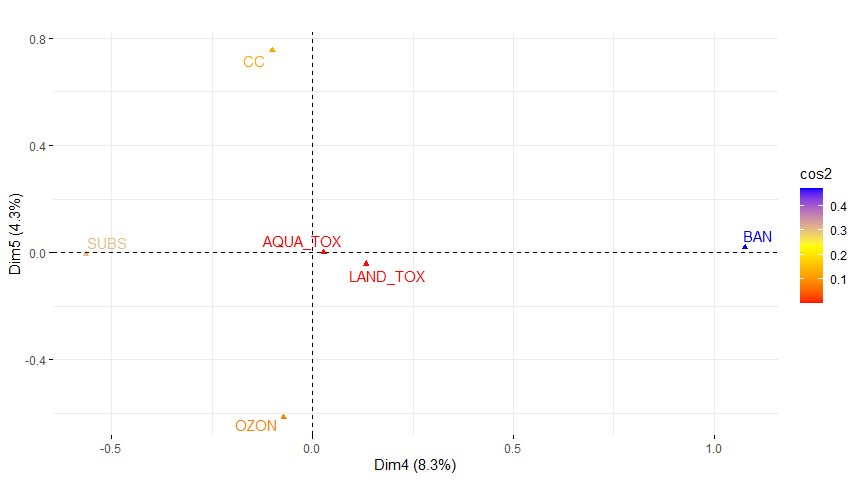


*Figure S1 Biplots for CA of the factors indicating which environmental harm the substances cause and whether they are banned or not or have close substitutes or not. 209 out of 217 substances included. Dim 4 and 5. The chi-square test rejects independence between rows and columns at 1% level. The color bar to the right shows the cosine square (cos2), and higher numbers (blue colors) indicate that the variable is well represented in the factor map.*

**Supplementary figure S2:**


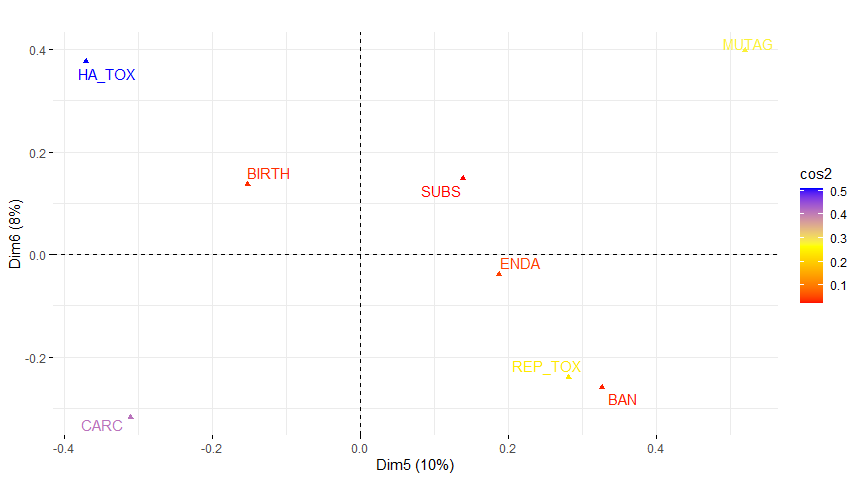


*Panel A*

*
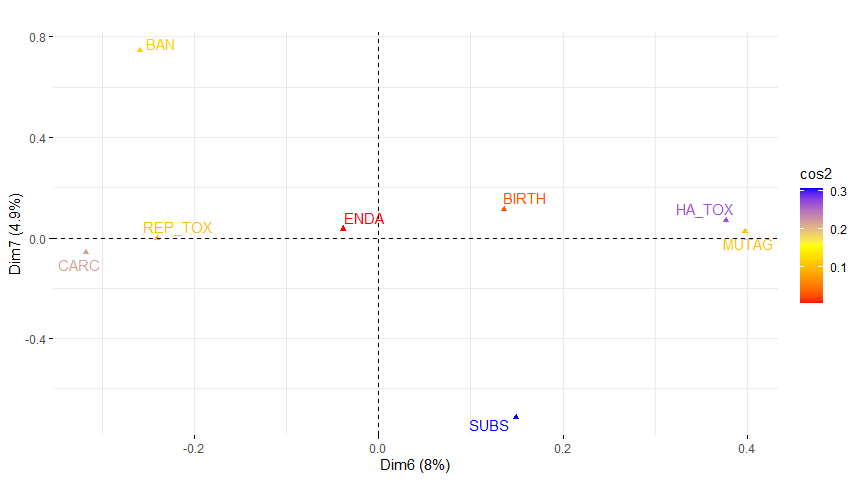
*

*Panel B*

*Figure S2 Biplot for CA of the factors indicating which human harm the substances cause and whether they are banned or not or have close substitutes or not. 217 substances included. Dim 5 and 6 in panel A, Dim 6 and 7 in panel B. The chi-square test rejects independence between rows and columns at 1% level. The color bar to the right shows the cosine square (cos2), and higher numbers (blue colors) indicate that the variable is well represented in the factor map.*
